# Part-Based Obstacle Detection Using a Multiple Output Neural Network

**DOI:** 10.3390/s22124312

**Published:** 2022-06-07

**Authors:** Razvan Itu, Radu Danescu

**Affiliations:** Computer Science Department, Technical University of Cluj-Napoca, St. Memorandumului 28, 400114 Cluj-Napoca, Romania; radu.danescu@cs.utcluj.ro

**Keywords:** driver assistance, CNNs, monocular vision, semantic segmentation, obstacle detection, instance segmentation, vanishing point

## Abstract

Detecting the objects surrounding a moving vehicle is essential for autonomous driving and for any kind of advanced driving assistance system; such a system can also be used for analyzing the surrounding traffic as the vehicle moves. The most popular techniques for object detection are based on image processing; in recent years, they have become increasingly focused on artificial intelligence. Systems using monocular vision are increasingly popular for driving assistance, as they do not require complex calibration and setup. The lack of three-dimensional data is compensated for by the efficient and accurate classification of the input image pixels. The detected objects are usually identified as cuboids in the 3D space, or as rectangles in the image space. Recently, instance segmentation techniques have been developed that are able to identify the freeform set of pixels that form an individual object, using complex convolutional neural networks (CNNs). This paper presents an alternative to these instance segmentation networks, combining much simpler semantic segmentation networks with light, geometrical post-processing techniques, to achieve instance segmentation results. The semantic segmentation network produces four semantic labels that identify the quarters of the individual objects: top left, top right, bottom left, and bottom right. These pixels are grouped into connected regions, based on their proximity and their position with respect to the whole object. Each quarter is used to generate a complete object hypothesis, which is then scored according to object pixel fitness. The individual homogeneous regions extracted from the labeled pixels are then assigned to the best-fitted rectangles, leading to complete and freeform identification of the pixels of individual objects. The accuracy is similar to instance segmentation-based methods but with reduced complexity in terms of trainable parameters, which leads to a reduced demand for computational resources.

## 1. Introduction

Artificial intelligence-based image processing has become popular in recent years and has improved the development of solutions in multiple fields, most notably in those that rely on computer-based vision. Deep learning systems feature in various real-life applications, making use of large publicly available datasets and of the increased processing power now available. Most notably, deep learning-based computer vision has been used to predict relevant information, with potential uses in various fields, such as medical imaging, piloting autonomous vehicles, robots or platforms, surveillance, and so on. For the prediction task, much of the work has been focused on developing convolutional neural networks (CNNs) that usually outperform the traditional image-processing algorithms.

Of all the possible uses for CNN-based computer vision, the field of automotive applications, which not only includes autonomous vehicles but also other advanced driving assistance systems, has benefited from special attention in recent years. This field is significant from the computer vision point of view because the environment to be perceived is dynamic, unpredictable, and complex, and the image perception must be performed in real time using limited computer architectures.

While there is a vast range of sensor systems available for autonomous vehicles, including laser scanners, radar, sonar, or stereo camera setups, a system that uses a single camera is much easier to set up and calibrate, but it can still extract a vast amount of information.

By analyzing the image provided by a single camera, a computer system can extract generic area information, such as the free-space areas in front of the vehicle that can be traversed or the obstacle areas that must be avoided, or it can extract information about individual objects. This generic area information can be used for basic collision avoidance or even for limited autonomy, such as lane-keeping or platooning, and the identification of individual objects helps in tracking them and predicting their intentions, or in classifying them, to better estimate the risks and vulnerabilities.

Both area perception and individual object perception functions can be achieved by CNNs. Identifying free space and obstacle areas can be achieved by using CNNs for semantic segmentation, which labels each pixel of the image with a class-based label; identifying individual objects can be achieved using instance segmentation CNNs.

In the past few years, much research has been proposed to improve semantic segmentation network architectures and to achieve accurate instance segmentation. The instance segmentation methods are a step ahead of the semantic methods. Semantic segmentation neural networks are faster but less complex. Semantic segmentation produces a label image with pixel-level information regarding the object’s class, whereas instance segmentation takes the semantic segmentation one step further to provide identifiers for individual object instances. To produce individual object instances, instance segmentation methods usually predict the object’s bounding box and then perform segmentation for each predicted bounding box. This introduces dependency in the network regarding the quality of the bounding box prediction. In addition, instance segmentation methods have proven to be time-consuming, and most solutions based on CNNs focus on performance, rather than prediction speed. Real-time capability is usually limited to object detection neural networks, which are mostly limited to predicting two-dimensional (2D) bounding boxes.

The work presented in this paper addresses the need for real-time instance segmentation with a reduced demand for resources. The core of our solution is a multiple output semantic segmentation network, which, while not able to identify individual objects, will produce object part information that is easily exploited by a low-complexity geometry-based algorithm. This combination of semantic segmentation and the geometric post-analysis of the semantic segmentation results is able to achieve the real-time identification of individual objects at the pixel level, with a significant reduction in the required resources when compared with instance segmentation networks.

In this paper, we propose a convolutional neural network that is trained to perform multiple prediction tasks, along with a geometry-based post-processing step to extract additional relevant data. The multi-output CNN prediction is achieved using a single encoder module and multiple decoders. The encoder part will extract the relevant features from the input images that are fed into the CNN and is usually referred to as the “backbone” network. The decoders will make use of these features to generate the predictions (outputs) of the network and are usually called network “heads”.

The presented CNN architecture is able to produce three different output heads related to the road traffic scene: the obstacle parts for instance segmentation (the top-left, top-right, bottom-left, and bottom-right quarters, which will be used later by the clustering algorithm), the semantic segmentation of the scene for detecting the drivable road area, and the vanishing point of the image. The backbone network (the encoder) features convolution layers that are used by the semantic segmentation head in order to construct images of the scene using the extracted features by using various upsampling layers and direct connections with the encoder.

The first head outputs the semantic segmentation of the object parts, which will be clustered using the algorithm presented in Section 3.4 to produce the instance segmentation results.

The second head produces the obstacle vs. free area semantic segmentation of the scene; this result is used as a mask to refine the object regions after they are clustered. The free space segmentation result can also be used as a standalone result for simpler driving assistance applications, such as collision avoidance.

The third head produces the gradient voting maps for vanishing point computation, a result that can be used for automatic camera orientation calibration and, therefore, for estimating the three-dimensional (3D) position of the objects detected by the previous two heads. This step is beyond the scope of this paper.

Thus, our proposed solution achieves obstacle reconstruction and individual instance extraction. We were able to perform semantic segmentation of the road traffic scene, detect obstacles and extract instances, and determine the vanishing point in an efficient manner, with results that were comparable to the ones produced by instance segmentation networks without the added memory requirements, high computational requirements, and network complexity.

The multi-output neural network is trained using the existing publicly available datasets. The training process is fully automated and does not require any annotation work, as the object quarters are extracted from labeled instances by our training pre-processing tool.

The main contributions of this work are the following:

1. A multiple-head network architecture that is able to produce the geometric parts of the objects, which can be then grouped into individual instances, free and occupied pixel classification for results refinement, and voting maps for vanishing point computation;
2. A lightweight object-clustering algorithm, based on the results of the multiple-head semantic segmentation network;
3. An automated training solution for the multiple-head network, which uses publicly available databases without the need for manual annotation of the object parts.

## 2. Related Work

### 2.1. Multi-Task Deep Learning

Deep learning-based solutions have been widely used for different tasks in recent years, most notably in approaches based on convolutional neural networks that are used for the classification of images [1], semantic image segmentation [2,3,4], object detection [5,6], or even tracking [7,8]. Different methods using deep learning for autonomous vehicles and driving have been presented elsewhere, in a survey [9]. In this paper, we focus on camera-based image processing; work using active sensors and sensor fusion has been also presented elsewhere and represents an active field of study [10,11].

Recent developments have been proposed in order to provide artificial neural networks that are able to perform multiple prediction tasks. The advantage of these solutions is that they improve computational efficiency and have a reduced memory footprint; however, the main drawback is that they are harder to train as they rely heavily on accurate and robust training datasets, which are laborious to produce. The multiple predictions of a single network are harder to inspect and debug. Still, multi-output neural networks have been developed; they proved to be scalable and easily expandable, meaning that an existing multi-task network can be expanded to predict new information as needed, including optical flow or tracking, scene depth, and so on. This is accomplished by reusing the network’s shared extracted features. Training multi-output networks can sometimes be challenging, due to the fact that the various outputs require different loss functions that need to be weighted. This process can be time-consuming and requires fine-tuning. Multi-task artificial networks improve learning by sharing the specific features of each task.

MultiNet [12,13] is a network that performs multiple predictions, whereas the work presented in [14] refers to a network that uses the YUV color space for the input images and can also produce multiple predictions. Elsewhere, a network that predicts semantic, instance, and depth information from a traffic scene has been presented in [15].

### 2.2. Object Detection

In the context of autonomous vehicles and driving, detecting objects can be performed with images acquired using a single camera [16] or a stereo-vision setup [17]. The detection task can also be achieved by using multiple input sources and sensorial data, such as lidar [10,18] or radar [19]. An overview of the multiple sensors used in autonomous vehicles is presented in [20]. The accuracy and robustness of a self-driving vehicle can be increased by using multiple sensors and fusing the data, but the main drawback is that these sensors require specific calibration and synchronization. The calibration and fusion of data add complexity to such a system, as well as additional costs.

One of the most convenient and affordable sources of information is the single camera, which is able to produce grayscale or color image streams. Single-camera systems are easy to set up and calibrate, and the lack of 3D information can be compensated for by increasingly complex and accurate algorithms.

Traditional object detectors have used image-processing-based algorithms, such as Viola-Jones [21] or a histogram of oriented gradients [22], whereas the modern object detection approaches are based on artificial learning, more specifically, convolutional neural networks. Some approaches fuse traditional and deep-learning methods for object detection [23]. Detecting objects is an extension of object classification, with the main aim of estimating the localization of all instances of certain objects from an input image. Recent detection methods that are based on artificial neural networks feature two main modules: a pre-trained backbone network and a head that outputs the object’s class, along with its bounding box coordinates. The backbone networks are used to extract the relevant features from the input images. Detection neural networks are trained using large amounts of labeled data; the detection process is implemented either in a single-stage (one-stage) or a two-stage approach.

Single-stage, also called one-stage, detectors localize objects and classify them in a single shot, by performing a regression of the bounding boxes, using predefined boxes and keypoints with multiple aspect ratios and scales.

Yolo [5], with its variations and improvements, and Single-Shot Detector (SSD) [6] are examples of one-stage detectors using CNNs. The authors of Yolo treated the detection problem as a regression problem, meaning that the CNN predicts the image pixels as the object, along with its bounding box coordinates and its class. Drawbacks regarding the localization of small objects from the original paper were fixed in the newer versions of Yolo [24], as well as by using different backbone networks, such as DarkNet [25]. Single-Shot Multibox Detector (SSD) used VGG-Net [26] as the backbone initially and matched the accuracy of some two-stage detectors. Limitations regarding small objects were fixed using ResNet [1] as the backbone. One-stage detectors usually achieve a very high frame rate, performing the detection in real time.

Two-stage detectors feature a separate module for region proposals, meaning that they are more complex in design and require more time for predictions. In the first step, the two-stage detectors generate regions of interest from the input image and use them in the second step to regress the results to object classes and bounding boxes. Usually, they offer better accuracy but lack real-time performance.

R-CNN [27], and its further improvements (Faster R-CNN [28]), represent two-stage detectors that use CNNs to classify and locate objects within images. The input image is fed into a region proposal module that outputs object predictions (candidates), which are then used in a second CNN module that extracts the object class and bounding box. These networks are usually more complex than one-stage detectors and need greater computational time for predictions. Mask R-CNN [29] extends the previous two-stage detectors, also performing instance segmentation by adding a mask head parallel to the bounding box and classification head. This network approach features good accuracy but lacks real-time performance. However, Mask R-CNN has remained one of the fastest networks for instance segmentation with challenging datasets. Other networks have been proposed that are faster and make use of techniques from object detectors, applying them to instance segmentation: Mask-YOLO [30] and YOLACT [31]. The YOLACT network divides the instance segmentation task into two parallel tasks to achieve real-time performance. A comprehensive survey regarding 2D image segmentation techniques using deep learning is presented in [32].

## 3. Solution Description

**Figure 1:**
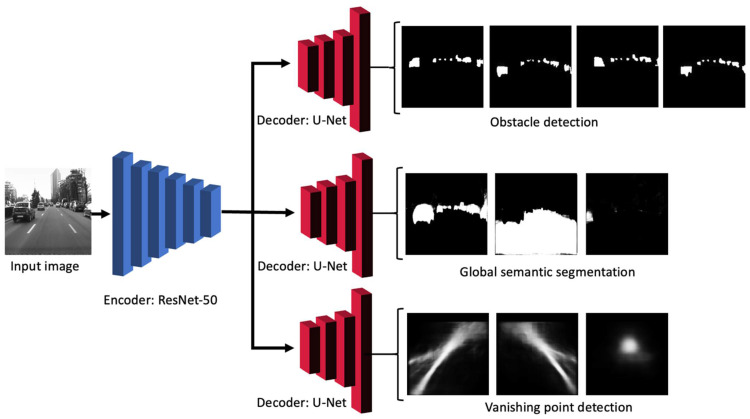
The multiple-output neural network architecture.

The core of the solution is the multiple-head artificial neural network, based on an encoder–decoder structure that uses a color image as input, whereas the output consists of multiple prediction modules: an obstacle detection module, a semantic segmentation module, and a vanishing-point detection module. The input part, based on an encoder, will extract the proper and significant features from the input image, whereas the decoder, which is based on semantic segmentation solutions, will provide multiple predictions (outputs). We have trained each module independently and we have used the same loss function. The CNN architecture is presented in Figure 1.

The detection module will identify individual objects or obstacles from the road scene, including vehicles, trucks, buses, cyclists, pedestrians, etc. This is based on semantic segmentation approaches; more specifically, on a modified U-Net CNN decoder module. The method of extracting the individual object instances is unique, meaning that we made use of the semantic segmentation CNN to label the image pixels with the corresponding object quarters (parts), instead of using it to label free space or specific obstacles. The labeled object parts were then grouped further into individual objects, using post-processing that took into account the proximity and position of the quarters as part of a full object. This approach simplifies the overall artificial network architecture by making use of the direct connections with the decoder’s layers. Another advantage of a multiple output network is that the encoder part is shared between the modules and the model can easily be extended, to predict other information. The detection module output features four layers (channels) that encode the binary status of the image pixels as being part of an object quarter (top left, top right, bottom left, bottom right). Using these parts, the algorithm described in Section 3.4 is able to extract the object instances as bounding boxes and as the labeled regions of pixels.

### 3.1. Feature Extraction

**Figure 2:**
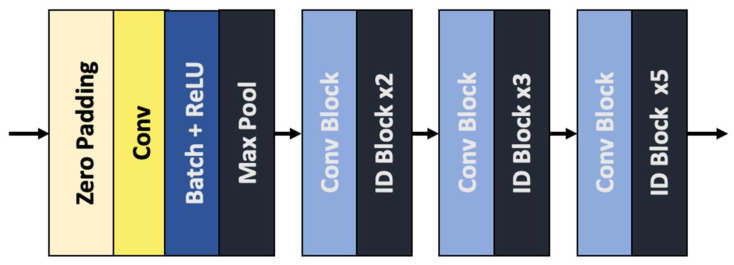
The layers of the ResNet-50 encoder.

The extraction of the relevant pixel-based features from the input images is achieved by the first part of the artificial network; its structure is based on the ResNet neural network architecture [1], which has proved to be very effective and is widely used. It gained recognition after it won the ImageNet competition in the past; it also introduced the concept of skip connections between the layers of the neural network. These connections were the novel part of the system and helped to improve the performance of the CNN. In this work, we make use of a modified version, which is called ResNet-50 (see Figure 2).

Each “conv block” is characterized by the following three operations: a 2D convolution, a batch normalization operation, and ReLU activation. These are performed three times, with a different number of filters and kernel sizes. This block is then combined with the result of running another 2D convolution. The “identity block” is similar, meaning that it has the same three operations that are also performed three times, but the skip connection is performed with the input tensor, rather than having the extra convolution at the end.

### 3.2. Decoder Structure

**Figure 3:**
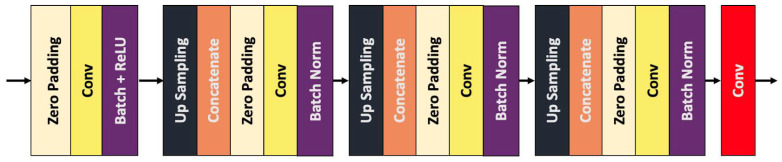
The layers of the proposed U-Net-based decoder.

The part responsible for decoding the extracted relevant features is the decoder. This is based on the well-known U-Net architecture [4], which is able to perform well, even with small training datasets. The structure of the decoder is illustrated in Figure 3. The concatenation operations are paired with the corresponding layers from the encoder part, whereas the output of the network is given by the final convolution operation.

The decoder makes use of multiple operations that are performed in order to obtain the desired output.

Our module features a central convolutional layer, followed by three upsampling layers. The central layer contains a 2D convolution operation and a batch normalization, followed by ReLU activation. The three upsampling layers have the following structure: 2D upsampling, concatenation (with the corresponding encoder layer), zero padding, 2D convolution, and a batch normalization operation. The final part from the semantic segmentation module has an additional convolution that represents the final segmentation output (the segmentation map), with the same number of layers as the desired number of predicted object classes.

**Figure 4:**
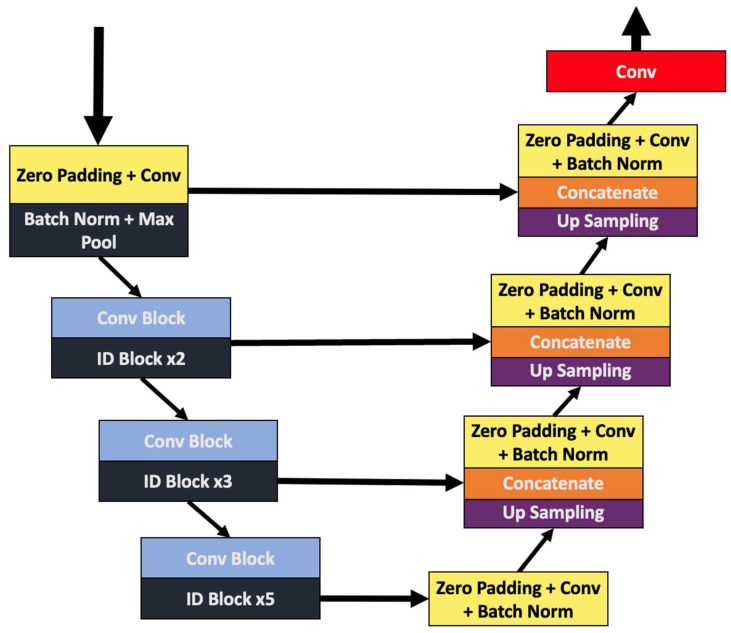
The CNN layers and the direct connections from the encoder (**left side**) to the decoder (**right side**), as illustrated.

The encoder-decoder structure of the artificial neural network is presented in Figure 4, where the concatenate connections are more clearly illustrated.

### 3.3. Global Semantic Segmentation

The neural network output (or heads) represents the decoder part that reconstructs the semantic segmentation and is based on the U-Net CNN. The network proposed by us features a center layer and three upsampling layers that are further concatenated with their correlated layers from the ResNet-based encoder, as described in the previous section.

**Figure 5:**
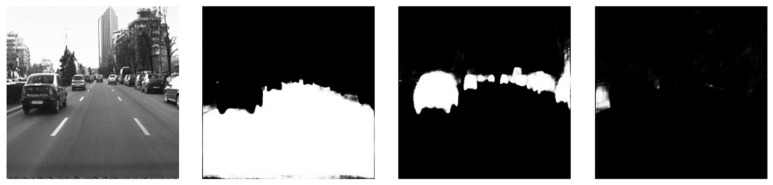
Examples of the global semantic segmentation output: the first image is the color input image of the road scene, while the second image represents the drivable road area, the third represents the dynamic objects, and the fourth image is static objects (sidewalk or fence).

The relevant features, extracted using the ResNet encoder, are used in the reconstruction layers of the U-Net decoder to produce an image with three channels, each channel representing the desired segmentation classes. In this proposed work, we predict three different classes: the road (free space), dynamic objects, and static objects. Therefore, the first output channel of the final convolutional layer will depict the drivable road area, while the second channel will represent the dynamic (moving) objects from the road, including pedestrians, cyclists, vehicles, buses, and trucks. The final channel of the output will be the static objects; more specifically, we chose sidewalks and the lane delimiters (road barriers or fences). An example is presented in Figure 5.

### 3.4. Obstacle Reconstruction Using Part-Based Semantic Segmentation

**Figure 6:**
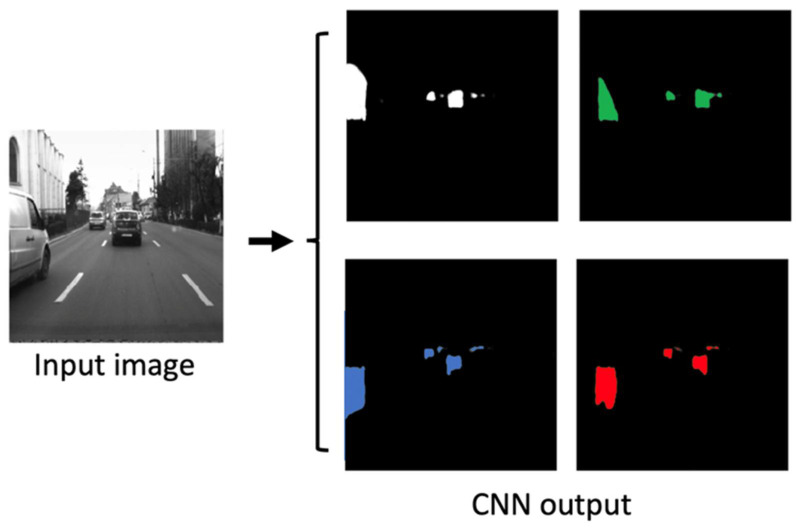
The artificial neural network, inferring the obstacle quarters.

The detected obstacles from the decoder module will be reconstructed using the methodology presented in this section. The CNN will provide an output wherein the obstacles are split into four individual quarters (parts), each represented by a binary channel of the output image (see Figure 6).

**Figure 7:**
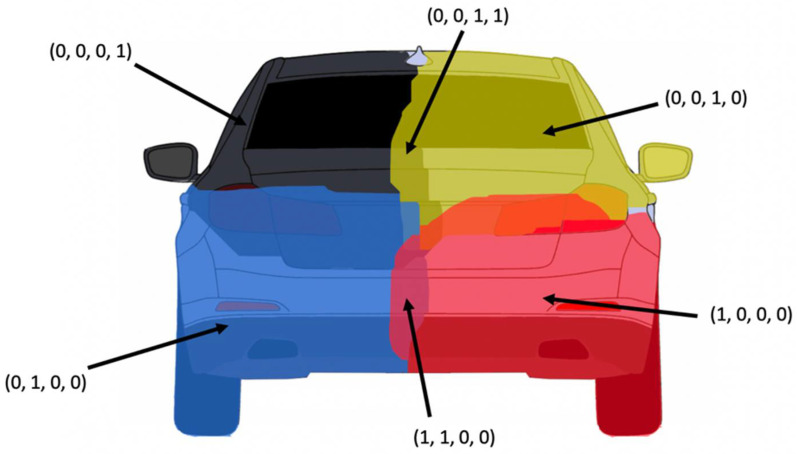
Object pixel coding, based on segmented quarters. Some pixels can belong to more than one quarter.

Based on the semantic segmentation results of the neural network, which labels each obstacle pixel with a “quarter” label, we have designed an algorithm to extract the individual objects. First, each pixel of the whole image space is labeled with a 4-bit code, each bit corresponding to a quarter that overlaps the pixel. Some pixels can be overlapped by more than one quarter, as the four semantic segmentation images produced by the CNN are not mutually exclusive (see Figure 7). The meaning of the four bits is as follows:

- Bit 0—top-left quarter;
- Bit 1—top-right quarter;
- Bit 2—bottom-left quarter;
- Bit 3—bottom-right quarter.

**Figure 8:**
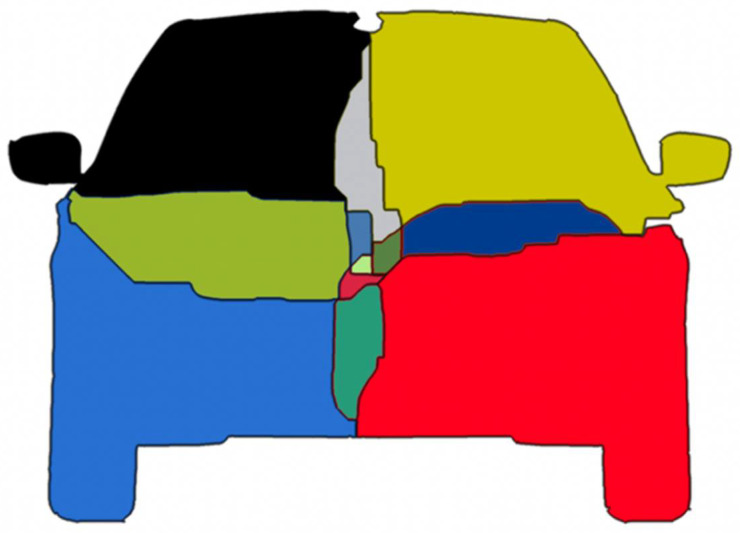
Labeled regions, based on connected pixels of the same quarter code.

The coded pixels will have a value of 0 if they belong to the free space (they are not obstacle points), or have a value between 1 and 15 if they belong to an object. For each of the 15-obstacle pixel values, a binary image will be generated, and the image will be labeled using the connected component labeling algorithm. The labels for each of the 15 images will be unique (the labels for image 2 will start from the maximum value of the labels of image 1, plus 1, and so on), meaning that at the end, they can be joined together into a common label image, as shown in Figure 8. The resulting regions are similar to the superpixels described in [11], but these have the added advantage that they are the result of semantic segmentation and have a high probability that each region belongs to a single object—they are grouped by meaning and not simply by color or texture properties, as in the case of superpixels.

The problem is now re-stated as the problem of assigning a unique object identifier to each region. Basically, the problem becomes a problem of region-grouping but, again, we can use the advantage of semantics because we can restrict the position of the region inside the whole object, based on the quarter codes.

**Figure 9:**
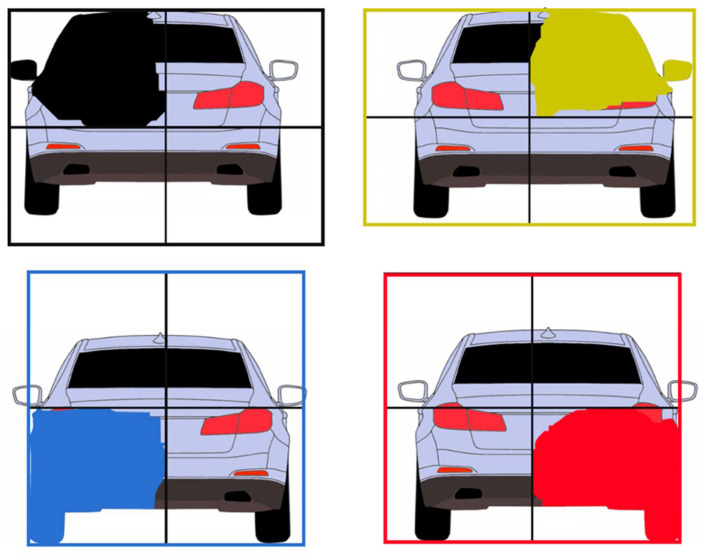
Generation of rectangle hypotheses, based on quarters.

The grouping of the regions will be based on generating multiple object hypotheses, based on the individual quarters. Because we have four types of quarters, we can generate a maximum of four hypotheses for each real-world object. Each quarter will generate a complete object by extending its size to cover the other three missing quarters, assuming that the quarters will be of a similar size. For example, a bottom-left quarter can be extended upwards and to the right to generate the possible complete object to which the quarter belongs. If the object is completely visible (not covered by another object and not at the edge of the image), four complete rectangular hypotheses will be generated, as seen in Figure 9.

**Figure 10:**
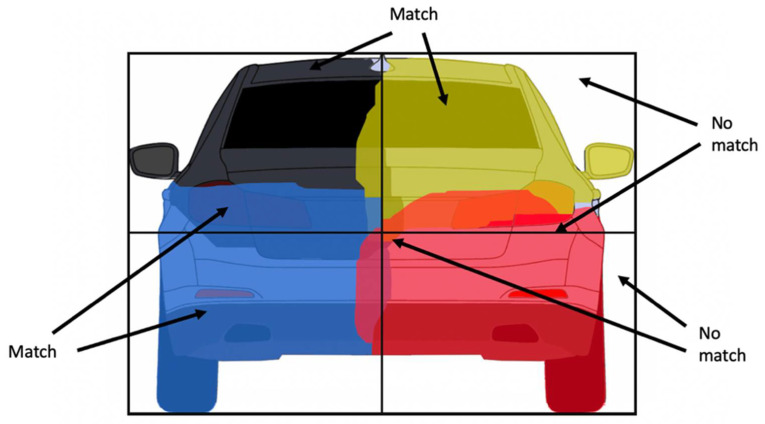
Computing the rectangle score, based on quarter matching.

Due to the fact that the hypotheses outnumber the objects by a factor of 4 to 1, the algorithm will compute a pixel fitness score for all hypotheses, so that the best-fitting one can be selected. The pixel score S(R) for each region R is computed by counting the pixels overlapping the region that fit with their corresponding quarter: the quarter defined by the region must correspond to the quarter label assigned to the pixel by the CNN-based semantic segmentation. When considering a matched pixel, we will consider the best-suited segmentation quarter label, so if a pixel has two labels (for example, a top-left label and a bottom-left label) and belongs to a top-left quarter of the hypothesis region, it is considered to be a match. This process is depicted in Figure 10.

The pixel score is then normalized with the region area, so all pixel scores S(R) will belong within the interval (0…1).

The next step of the algorithm is to establish the dependency relationships between the hypotheses. An overlapping score is computed between any two rectangles Ri and Rj. If the overlapping score exceeds a threshold of 0.5, and the pixel score of Rj is higher than the pixel score of Ri, the rectangle Ri will be labeled as being dependent on Rj. Basically, we assume that the region Ri depicts the same object as Rj but is a less adequate fit.

**Figure 11:**
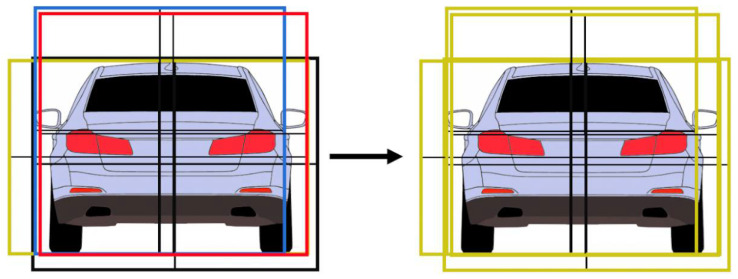
Re-labeling the rectangle hypothesis by the best-scored overlap.

This process is depicted in Figure 11. The four rectangles are generated by the pixel-derived quarters, but the best fit is the rectangle generated by the top-right quarter. Therefore, all the other rectangles are labeled as being dependent on the rectangle hypothesis generated by the top-right quarter.

Based on the rectangle hypotheses and their dependency relations, the individual regions presented in Figure 8 will receive the final label. This new label is the identity of the individual object of which the region is a part. 

For each region, the number of pixels overlapping every rectangle hypothesis is counted. All rectangles are considered in this step, even those that are in a dependency relationship. The process has two stages:

1. The rectangle that overlaps the most pixels of the individual region is selected.
2. If the rectangle is dependent on another rectangle (as seen in Figure 11), the label of the main rectangle is transferred to the individual region.

The final step of the algorithm is to label the pixels themselves with the identity of the object to which they belong. For this step, we simply transfer to the individual pixel the label of the region to which the pixel belongs, as this region has already been labeled with the identity of the object.

**Figure 12:**
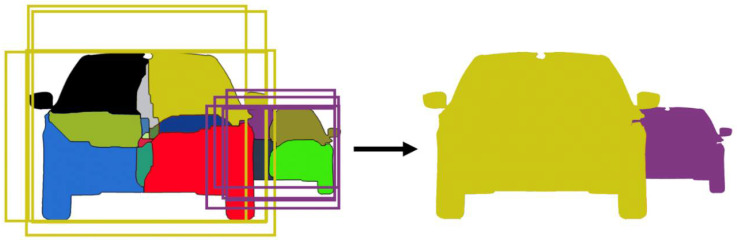
The final result, obtained by labeling the regions with the rectangle labels, then transferring the region labels to the individual pixels.

The entire process is illustrated in Figure 12, where two partially overlapping objects are presented, together with their final label. The complete processing steps are described in Algorithm 1.
**Algorithm 1** Instance segmentation**Input:** TL, TR, BL, BR—Top-left, top-right, bottom-left, and bottom-right binary images **Output:** L—image labeled with the individual object identifiers**1. Identification of connected regions from each of the four binary images:**  TL_Regions = ConnectedLabeling (TL)  TR_Regions = ConnectedLabeling (TR)  BL_Regions = ConnectedLabeling (BL)  BR_Regions = ConnectedLabeling (BR) **2. Creation of the composite type image:**  **For** each (x,y)   Composite(x,y) = BR(x,y) <<3 | BL(x,y) <<2 | TR(x,y) <<1 | TL(x,y) **3. Labeling the composite image:**  Regions = []  **For** type = 1 to 15   Regions = Regions U ConnectedLabeling (Composite = type) **4. Generating the hypothetical rectangles:**  TL_Rectangles = ExpandRectangle (TL_Regions)  TR_Rectangles = ExpandRectangle (TR_Regions)  BL_Rectangles = ExpandRectangle (BL_Regions)  BR_Rectangles = ExpandRectangle (BR_Regions)  R = TL_Rectangles U TR_Rectangles U BL_Rectangles U BR_Rectangles  S(R) = ComputeRectangleScores (R) **5. Labeling the rectangles**  **For** each rectangle Ri   LR (i) = i  **Repeat**  **For** i   **For** j     **If** Overlap (Ri, Rj) > 0.5 and S (Ri) > S (Rj)       LR(j) = LR (i)  **Until** no change of LR **6. Labeling the regions**  **For** each Reg in Regions   R = ArgMax R(Overlap (R, Reg))   L(Reg) = LR(R)

### 3.5. Object Refinement

**Figure 13:**
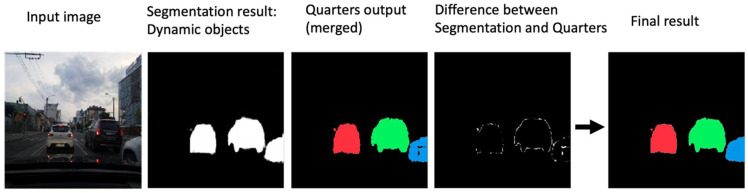
Fusing the semantic output with the quarters to improve object detection.

We provide an additional post-processing step in order to improve the quality of the detected objects. The dynamic objects from the global segmentation head (described in Section 3.3) are used to improve the four quarters from the object detection head. Therefore, the semantic output is used to refine the edges of the detected quarters. The process is depicted in Figure 13, where the global semantic segmentation dynamic objects (column 2) are combined with the quarters output (column 3).

### 3.6. Vanishing-Point Computation

**Figure 14:**
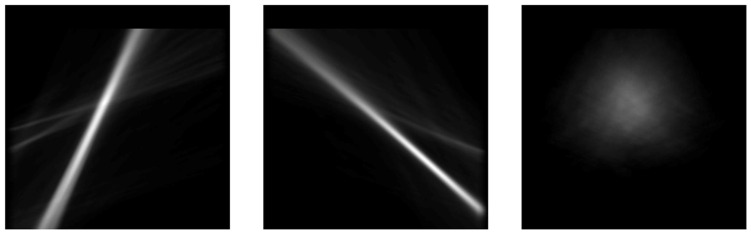
The vote-map features of the vanishing point: the left side features (**first image**), the right side features (**center**), and the multiplication result of the first two images (**right**).

Vanishing-point computation methods have been presented in the literature. Most methods are based either on line intersections or feature analysis or use artificial networks to predict the 2D point coordinates of the vanishing point [33]. In this work, we make use of the results of our previously published work [34] to produce the required training images for the neural network. Our previous paper presented an algorithm for detecting the vanishing point by computing the orientation and magnitude of the gradient that is used to generate three vote-map images. The first image contains the vote map of the features from the left side of the input image, the second image has the vote-map features from the right side of the input image, and the third image is actually the multiplication result of the first two images (left and right vote maps). Figure 14 presents an example of the three vote-map images. The voting maps can be computed using classic image processing techniques (by computing the orientation of the gradients) or can be extracted directly from a fully convolutional CNN, such as the one proposed in this paper.

The coordinates of the vanishing point can be computed using a sliding window on the third image, or by extracting the maximum from the third vote map image. Extracting the maximum takes an additional 0.09 ms on average to compute; therefore, we used this version. Extracting the VP coordinates can be achieved directly in an end-to-end manner via a CNN with a different architecture; however, in our case, we preferred to leverage the shared layers from the encoder, in order to produce the three vote-map images using semantic segmentation and then extract the coordinates from the third output layer. The other two predicted layers (the left and right vote maps) can be used as input for a lane detection system on marked roads, or as an additional step to validate the extracted vanishing-point coordinates. 

Computing the vanishing point is relevant, due to the fact that it can be used to compute the extrinsic camera parameters if we assume some geometric constraints (a flat road assumption and a small lens distortion). The pitch and yaw angles of the camera with respect to the world can be computed if the focal distance is also known (along with the image size), as we presented in [33].

## 4. Training the Multi-Output CNN

**Figure 15:**
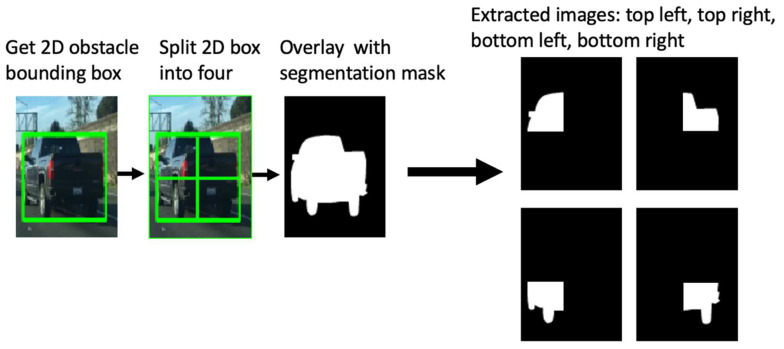
Extracting the obstacle quarters from the original segmentation mask and 2D obstacle bounding boxes. Image source [36].

Multi-task learning requires special datasets and databases for training. For the proposed solution, we have used three well-known datasets: CityScapes [35], the Berkeley Deep Drive (BDD) [36], and Mapillary [37]. All the images were processed in order to have the same size and aspect ratio; we also filtered the images to select only those that feature a large number of road pixels. After this selection, we processed the number of images used for training, as follows: 2759 images were taken from BDD, 2975 images from CityScapes, and 17,109 images from Mapillary. These databases contain relevant information for semantic segmentation and the bounding boxes of the obstacle objects. By using this data, we could extract the four obstacle quarters. The input images were split into four quarters, using the available bounding boxes of the objects. The next step consisted of masking each quarter with the semantic segmentation maps in order to generate the top-left, top-right, bottom-left, and bottom-right binary images of the individual object instances. The process is presented in Figure 15.

The prepared images from the datasets were also augmented during training. We used the techniques of random intensity and saturation adjustments in the HSV color space, as well as random image scaling and translation. 

The semantic segmentation module used binary cross-entropy and the Sorensen–Dice [38] loss function, which is a modified version of the intersect over union loss. The loss function for the obstacle detection was the same. The vanishing point module also used the same loss function as the semantic segmentation; generating the training data is described in Section 3.6 During the training process, each loss function can be configured with a different weight. We used the same weight for all three loss functions, having experimented with various settings.

Training the neural network was performed for a total of 500 epochs; it was executed on a system equipped with two Nvidia 1080 Ti GPUs that featured a total of 22 GB of memory. The patience parameter was set to 50 epochs; therefore, if the loss function did not improve for 50 epochs, the training process was stopped early. With this hardware, training one epoch took around 300 s, which means that a fully working multi-output CNN model can be obtained in less than 10 h (with early stopping). For our system, the ResNet-50 encoder was initialized with the weights from ResNet-50 and trained on ImageNet, which helped speed up the training process. We also tried freezing some layers during training, but this did not improve the final results.

## 5. Results and Evaluation

The same hardware setup was used for evaluating as well as training the model. The experimental setup is based on the Python programming language, the project being implemented using open-source deep learning software. The proposed multiple output network was evaluated, using not only existing popular datasets (as mentioned in Section 4) but also our own datasets [17,33]. 

For the semantic segmentation output head, the evaluation was performed using the CityScapes validation dataset; the results were published in our previous work [16], where we used the free road space in order to detect on-road obstacles and the segmented road surface was integrated into our monocular perception system, which is able to track the detected obstacles using a particle filter. For the road class, we obtained an IoU score of 0.90.

The obstacle detection output head that predicts the obstacle quarters represents the main information used for detecting the obstacles from the road traffic scene. Therefore, the obstacle detection module was also evaluated using the dataset presented in [17], which features road-traffic images captured with different camera systems from the ones used in the training datasets. For this dataset, the ground truth is considered to be the information obtained from a stereo-vision camera setup. We compared our results with our previously proposed system and previously published results in [39,40]. On our own dataset, which features over 1000 images of object-bounding boxes extracted from the stereo-vision data, we obtained an IoU score of 0.74. On the CityScapes validation dataset, we obtained an IoU score of 0.83 and, on the KITTI [41] dataset, a 0.70 IoU score. The results were also compared with a Yolo V3 [5] detector featuring a DarkNet backbone, which was trained on KITTI using the same loss as the initial Yolo paper. We also compared the results with a Yolo V3 detector with the same ResNet-50 backbone, trained on the same datasets as our proposed model. We have also compared our results with the bounding boxes from the Mask R-CNN and Yolact; the results are similar to ours, with minor differences (1–2% in favor of the Mask R-CNN or Yolact). The bounding box obstacle detection results are presented in Table 1.

The stereo-vision database from Table 1 used images acquired with a pair of cameras from which we used only the left image from the setup. The ground truth data was considered to be the result of a stereo-tracking algorithm [42]. The main advantage of a stereo-vision setup is that in the case of overlapping objects, they can be easily detected based on their depth information. Still, our proposed system, using a monocular camera setup, obtained a high accuracy. The evaluation in terms of complex city scenarios was proven to be highly accurate, especially using the CityScapes dataset. When testing on the KITTI database, our results were poorer due to the different aspect ratios of the dataset images. To address this issue, we had two possibilities: we either resized the KITTI image to fit the 256 × 256 pixels input of the CNN, or we cropped the KITTI image and then resized it. Both variants would affect our evaluation results; the first choice would produce a severe deformation of the objects from the traffic scene, which would highly impact the detection process, whereas the second choice (cropping the input image), would most likely prevent various objects from the scene being considered for detection. For this evaluation, we used the first option.

For the 2D bounding box (object detection) evaluation, we obtained a marginal improvement (1%) from previously published work [40], due to the image fusion of the output heads (as described in Section 3.5).

The pixel-wise evaluation of the detection was also performed. We evaluated the instance segmentation on the CityScapes dataset and compared the results with an R-CNN and Yolact, tested on the same test data from the dataset. The results are presented in Table 2.

**Figure 16:**
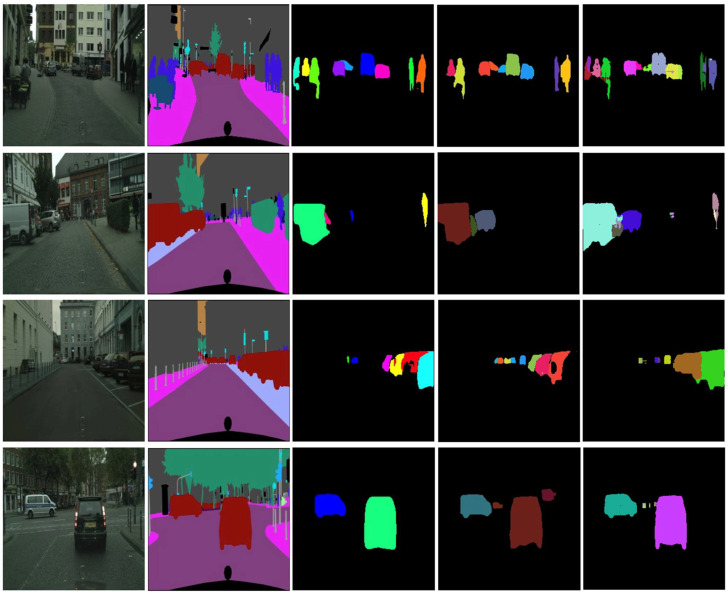
Comparison between Mask R-CNN, Yolact, and our method: the first column represents the input image, the second column shows the ground truth instances, the third column shows the Mask R-CNN results, the fourth column shows the Yolact results, and the fifth column represents our results.

We also present some of the qualitative results of our proposed network, versus Yolact and R-CNN, in Figure 16. The Mask R-CNN would extract well-defined obstacle edges that were more refined, but, in some cases, this method might miss objects (as can be seen in the second row of Figure 16). Yolact predictions are sometimes missing some objects from the scene, as seen in the second or third row of Figure 16; the Yolact solution also seems to predict false positives (fourth row of Figure 16).

The popular Mask R-CNN network has a total of 64 million parameters, while Yolact has 50 million parameters; Yolo V3, with a Darknet backbone, features 41 million parameters, while our U-Net- and ResNet-based model features 32 million parameters in total for the three output heads (semantic segmentation, vanishing point, and obstacle quarters). The total prediction time for all three outputs was 0.043 s on average, whereas for the Mask R-CNN, the prediction time was 0.23 s and, for Yolo V3, it was 0.052 s. The Yolact network predicted the output in 0.041 s. If we removed the vanishing point output head, the network was reduced to 24 million parameters and the prediction time was then 0.034 s. If we further removed the semantic segmentation output head, we ended up with a 16-million-parameter network with a prediction time of 0.026 s.

All the results are presented in Table 3 and the tests were performed on a desktop system, equipped with an Intel i7 CPU and two Nvidia 1080 Ti GPU graphic boards that were used for both training and prediction (evaluation).

The prediction time for all three output heads was similar to the one from Yolo V3, which only features obstacle detection (0.057 vs. 0.052 s, on average, on the same input test images). The total computational time for our system was higher, due to the extra post-processing required for the obstacle quarter grouping, labeling, and, finally, bounding box extraction (0.014 s); however, these steps were executed on a single CPU core and were not optimized. Nevertheless, the processing times were comparable.

**Figure 17:**
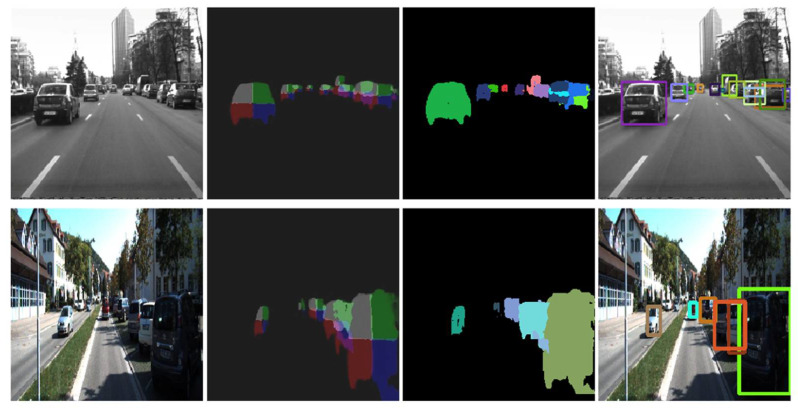

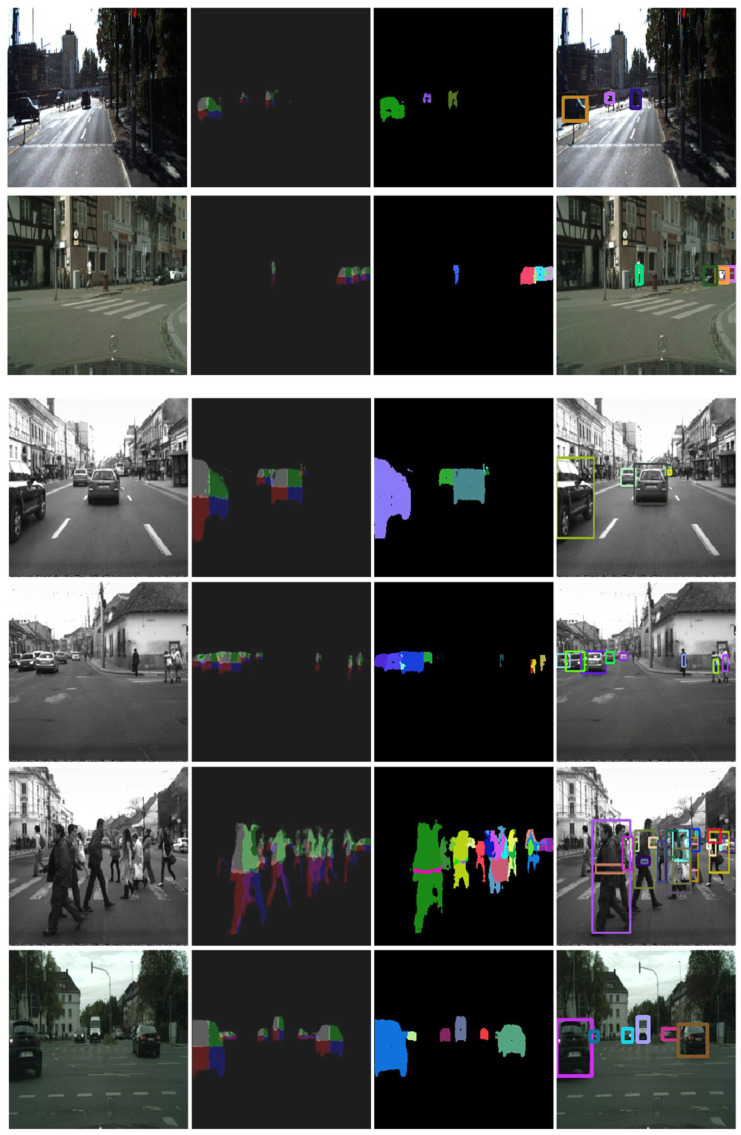
The first column represents the input image, while the second column is the quarter prediction (with the CNN output merged), the third column represents the labeling output, and the fourth column is the resulting bounding boxes of the individual obstacles.

The prediction and post-processing results of the proposed system, in various scenarios, are illustrated in Figure 17. A video with the results on the stereo-vision dataset is available at: https://vimeo.com/694007992 (accessed on 20 April 2022).

The third output head represents the vanishing-point prediction. The vanishing-point coordinates are extracted from the CNN-predicted mask, which takes an additional 0.09 ms on average. The results are presented in Table 4, as already published in our previous work [39].

The evaluation presented in Table 4 was performed using the CityScapes test set and the dataset from [33]. The metric used was NormDist, which represents the RMSE pixel error, divided by the image diagonal.

## 6. Conclusions

In this paper, we propose a solution that is capable of accurately detecting on-road obstacles and their actual instances in various road-traffic scenarios. We achieved this by leveraging encoder–decoder-based artificial networks and geometry-based computer vision algorithms. The information about object parts was extracted using semantic segmentation networks and then used in a low-complexity clustering algorithm. Therefore, our proposed solution is capable of detecting individual objects, even if they are partly occluded or are in close contact. The multi-output CNN model, together with the post-processing algorithm, represents a different approach to the traditional object segmentation and detection problem. The system is also lighter in terms of computational demand and is easier to train, while also having an accuracy comparable with much more complex artificial neural networks. We have presented a unique approach to detecting object instances, along with the semantic segmentation of the scene. In this approach, we fuse the two prediction outputs to obtain better object instances; we also predict information regarding the vanishing point that can be used later to compute the extrinsic camera parameters. In conclusion, the proposed approach features a lower number of parameters than in similar published work, has similar or better performance than previous approaches when evaluating instances and the 2D bounding boxes, and is easy to train and deploy. The proposed solution can be used in real-time systems with a single camera to predict individual obstacle instances in road-traffic scenarios, while also predicting the vanishing point, which can be used for the self-calibration of the camera. Our proposed solution has been evaluated using well-established performance indicators on publicly available datasets and our own acquired database.

Future work will include using these results along with a tracker, in order to estimate the trajectories of the road participants. The idea is to make use of multiple consecutive frames, in order to better determine partly or fully occluded objects from the road scene. This future work will also include exploring other prediction outputs for the CNN model, such as depth or optical flow.

## Figures and Tables

**Table 1:** Bounding box evaluation.

Solution	Stereo Dataset (IoU)	CityScapes Dataset (IoU)	KITTI Dataset (IoU)
MONet V1 with YOLO head [39]	0.64	0.63	-
MONet V1 [39]	0.74	0.80	0.55
Proposed system	0.74	0.83	0.70
Mask R-CNN [29]	0.74	0.84	0.68
DarkNet with YOLO V3 [25]	0.56	0.57	0.88
Yolact [31]	0.73	0.84	0.68

**Table 2:** Instance segmentation evaluation.

Solution	Accuracy	Precision	Recall	F1 Score
Mask R-CNN [29]	0.91	0.93	0.24	0.34
Yolact [31]	0.87	0.67	0.17	0.24
Proposed system	0.93	0.94	0.44	0.57

**Table 3:** CNN comparisons.

Solution	Total Number of Parameters (All Output Heads)	CNN Prediction Time (Seconds)	Total Computation Time (Predict + Extract Bounding Boxes) in Seconds
MONet V1, with YOLO obstacle detection head	42 million	0.062	0.063
MONet V1 [39]	32 million	0.043	0.093
MONet V2 [40]	32 million	0.043	0.061
Proposed system	32 million	0.043	0.057
Proposed system, without vanishing point head	24 million	0.034	0.048
Proposed system, without vanishing point head and without semantic head	16 million	0.026	0.040
DarkNet, with YOLO V3 [25]	41 million	0.052	0.052
Mask R-CNN [29]	64 million	0.23	0.23
Yolact [31]	50 million	0.041	0.044

**Table 4:** Vanishing point evaluation.

Solution	CityScapes (NormDist)	VP Highway (NormDist)	VP City (NormDist)
MONet V1 [39]	-	0.050	0.026
Proposed system	0.016	0.013	0.018

## Data Availability

Not applicable.

## References

[1] He K., Zhang X., Ren S., Sun J. Deep residual learning for image recognition. Proceedings of the 2016 IEEE Conference on Computer Vision and Pattern Recognition (CVPR).

[2] Zhong Z., Li J., Cui W., Jiang H. Fully convolutional networks for building and road extraction: Preliminary results. Proceedings of the 2016 IEEE International Geoscience and Remote Sensing Symposium (IGARSS).

[3] Badrinarayanan V., Kendall A., Cipolla R. (2017). SegNet: A Deep Convolutional Encoder-Decoder Architecture for Image Segmentation. IEEE Trans. Pattern Anal. Mach. Intell..

[4] Ronneberger O., Fischer P., Brox T. U-Net: Convolutional Networks for Biomedical Image Segmentation. Proceedings of the 18th International Conference on Medical Image Computing and Computer-Assisted Intervention.

[5] Redmon J., Divvala S., Girshick R., Farhadi A. You only look once: Unified, real-time object detection. Proceedings of the 2016 IEEE Conference on Computer Vision and Pattern Recognition (CVPR).

[6] Liu W., Anguelov D., Erhan D., Szegedy C., Reed S., Fu C.-Y., Berg A.C. (2016). SSD: Single shot MultiBox detector. arXiv.

[7] David H., Sebastian T., Silvio S. (2016). Learning to Track at 100 FPS with Deep Regression Networks. arXiv.

[8] Hu H.-N., Cai Q.-Z., Wang D., Lin J., Sun M., Krahenbuhl P., Darrell T., Yu F. (2018). Joint Monocular 3D Vehicle Detection and Tracking. arXiv.

[9] Ni J., Chen Y., Chen Y., Zhu J., Ali D., Cao W. (2020). A Survey on Theories and Applications for Self-Driving Cars Based on Deep Learning Methods. Appl. Sci..

[10] Muresan M.P., Giosan I., Nedevschi S. (2020). Stabilization and Validation of 3D Object Position Using Multimodal Sensor Fusion and Semantic Segmentation. Sensors.

[11] Shahian Jahromi B., Tulabandhula T., Cetin S. (2019). Real-Time Hybrid Multi-Sensor Fusion Framework for Perception in Autonomous Vehicles. Sensors.

[12] Boulay T. (2019). YUVMultiNet: Real-time YUV multi-task CNN for autonomous driving. arXiv.

[13] Teichmann M. (2016). MultiNet: Real-time Joint Semantic Reasoning for Autonomous Driving. arXiv.

[14] Sistu G., Leang I., Yogamani S. (2019). Real-time Joint Object Detection and Semantic Segmentation Network for Automated Driving. arXiv.

[15] Kendall A., Gal Y., Cipolla R. (2018). Multi-task learning using uncertainty to weigh losses for scene geometry and semantics. arXiv.

[16] Itu R., Danescu R.G. (2020). A Self-Calibrating Probabilistic Framework for 3D Environment Perception Using Monocular Vision. Sensors.

[17] Nedevschi S., Danescu R., Frentiu D., Marita T., Oniga F., Pocol C., Schmidt R., Graf T. High accuracy stereo vision system for far distance obstacle detection. Proceedings of the IEEE Intelligent Vehicles Symposium.

[18] Kumar G.A., Lee J.H., Hwang J., Park J., Youn S.H., Kwon S. (2020). LiDAR and Camera Fusion Approach for Object Distance Estimation in Self-Driving Vehicles. Symmetry.

[19] Song W., Yang Y., Fu M., Qiu F., Wang M. (2018). Real-Time Obstacles Detection and Status Classification for Collision Warning in a Vehicle Active Safety System. IEEE Trans. Intell. Transp. Syst..

[20] Yeong D.J., Velasco-Hernandez G., Barry J., Walsh J. (2021). Sensor and Sensor Fusion Technology in Autonomous Vehicles: A Review. Sensors.

[21] Viola P., Jones M. Rapid object detection using a boosted cascade of simple features. Proceedings of the 2001 IEEE Computer Society Conference on Computer Vision and Pattern Recognition, CVPR 2001.

[22] Dalal N., Triggs B. Histograms of oriented gradients for human detection. Proceedings of the 2005 IEEE Computer Society Conference on Computer Vision and Pattern Recognition, CVPR 2005.

[23] Gao F., Wang C., Li C. (2020). A Combined Object Detection Method with Application to Pedestrian Detection. IEEE Access.

[24] Redmon J., Farhadi A. (2016). YOLO9000: Better, faster, stronger. arXiv.

[25] Redmon J. Darknet: Open Source Neural Networks in c. http://pjreddie.com/darknet/.

[26] Simonyan K., Zisserman A. (2014). Very deep convolutional networks for large-scale image recognition. arXiv.

[27] Girshick R., Donahue J., Darrell T., Malik J. (2014). Rich feature hierarchies for accurate object detection and semantic segmentation. arXiv.

[28] Ren S., He K., Girshick R., Sun J. (2016). Faster r-cnn: Towards real-time object detection with region proposal networks. arXiv.

[29] He K., Gkioxari G., Dollar P., Girshick R. Mask R-CNN. Proceedings of the 2017 IEEE International Conference on Computer Vision (ICCV).

[30] Liu H. Mask-YOLO: Efficient Instance-level Segmentation Network Based on YOLO-V2. https://ansleliu.github.io/MaskYOLO.html.

[31] Bolya D., Zhou C., Xiao F., Lee Y.J. (2019). YOLACT: Real-time Instance Segmentation. arXiv.

[32] Irem Ulku I., Akagunduz E. (2022). A Survey on Deep Learning-based Architectures for Semantic Segmentation on 2D images. arXiv.

[33] Itu R., Borza D., Danescu R. Automatic extrinsic camera parameters calibration using Convolutional Neural Networks. Proceedings of the 2017 IEEE 13th International Conference on Intelligent Computer Communication and Processing (ICCP 2017).

[34] Danescu R., Itu R. Camera Calibration for CNN-based Generic Obstacle Detection. Proceedings of the 19th EPIA Conference on Artificial Intelligence.

[35] Cordts M., Omran M., Ramos S., Rehfeld T., Enzweiler M., Benenson R., Franke U., Roth S., Schiele B. The Cityscapes Dataset for Semantic Urban Scene Understanding. Proceedings of the Computer Vision and Pattern Recognition.

[36] Yu F., Xian W., Chen Y., Liu F., Liao M., Madhavan V., Darrell T. (2018). BDD100K: A Diverse Driving Video Database with Scalable Annotation Tooling. arXiv.

[37] Neuhold G., Ollmann T., Bulò S.R., Kontschieder P. The Mapillary Vistas Dataset for Semantic Understanding of Street Scenes. Proceedings of the 2017 IEEE International Conference on Computer Vision (ICCV).

[38] Sorensen T. (1948). A method of establishing groups of equal amplitude in plant sociology based on similarity of species and its application to analyses of the vegetation on Danish commons. K. Dan. Vidensk. Selsk..

[39] Itu R., Danescu R. MONet—Multiple Output Network for Driver Assistance Systems Based on a Monocular Camera. Proceedings of the 2020 IEEE 16th International Conference on Intelligent Computer Communication and Processing (ICCP 2020).

[40] Itu R., Danescu R. Object detection using part based semantic segmentation. Proceedings of the 2021 IEEE 17th International Conference on Intelligent Computer Communication and Processing (ICCP 2021).

[41] Geiger A., Lenz P., Urtasun R. Are we ready for Autonomous Driving? The KITTI Vision Benchmark Suite. Proceedings of the 2012 IEEE Conference on Computer Vision and Pattern Recognition.

[42] Danescu R., Pantilie C., Oniga F., Nedevschi S. (2012). Particle Grid Tracking System for Stereovision Based Obstacle Perception in Driving Environments. IEEE Intell. Transp. Syst. Mag..

